# Does It Matter Who Writes Medical News Stories?

**DOI:** 10.1371/journal.pmed.1000323

**Published:** 2010-09-14

**Authors:** Amanda Wilson, Jane Robertson, Patrick McElduff, Alison Jones, David Henry

**Affiliations:** 1 The School of Medicine and Public Health, Faculty of Health, University of Newcastle, Waratah, Australia; 2 Centre for Clinical Epidemiology and Biostatistics, Faculty of Health, University of Newcastle, Newcastle, Australia; 3 School of Medicine, University of Western Sydney, Sydney, Australia; 4 The Institute for Clinical Evaluative Sciences, Toronto, Canada

## Abstract

David Henry and colleagues review Australian news stories over a five-year period to assess whether quality is associated with who wrote the story: a specialist health journalist or a non-specialist.

**Summary Points**

- The media can influence health literacy and health seeking behaviours, but few studies have looked at the quality of news stories. We examined whether experienced specialist health reporters write better stories than other categories of journalists
- We compared the quality of stories written by specialist and non-specialist journalists, and those sourced from major news organisations, in Australia from 2004–08.
- We found that it does matter who writes news stories that cover the benefits and harms of health care interventions. Stories written by specialist health journalists working for a single media outlet scored more highly than those written by less experienced writers.
- Our findings are important because this source of health literacy is currently under pressure as falling revenues threaten the future of the traditional media.

## Background

The news media have a crucial role in supporting health literacy, and multiple surveys have shown the extent to which the public relies on them for information about medical advances [1],[2]. However, the mainstream media are undergoing rapid and unprecedented change, with a shift from the traditional outlets (broadsheet newspapers and flagship current affairs programs) to online news services and blogs that are available free of charge. These online sources, and the more recent Web 2.0 activities (e.g., FaceBook and Twitter), still rely on the quality of the news coverage by the traditional media, which they frequently cite as information sources [3].

A number of recent studies have pointed to the poor and variable quality of many health stories in the mainstream media, particularly those covering new drugs and procedures [4],[5]. Some outlets are capable of producing excellent stories, but common flaws across all media include lack of attention to the quality of the research evidence, exaggerated estimates of benefits, inadequate coverage of potential harm, no information on the costs of new treatments and a failure to identify unbiased expert sources. Studies have revealed such deficiencies in Australia, Canada and the United States, with little evidence of improvement in reporting over the last five years [4]–[7]. The reasons for poor-quality journalism are complex, and include lack of specialised knowledge, time pressures on journalists, space limitations, the difficulty of accessing expert unbiased informants, and the desire of researchers, their institutions, and (sometimes) journals to exaggerate the significance of the research [8]–[11].

But what impact will the current financial pressures on the traditional media have on the already variable quality of medical news reporting? Should a newspaper editor faced with falling sales and advertising revenue retain the services of a specialised but more expensive medical journalist, who can interpret new scientific data and place it in a local context? Or will a non-specialist journalist do the job as well? Can medical news content be reliably imported from overseas media, or from news organisations such as Associated Press and Reuters? Here, we examine the question “does it matter who writes the stories?”

## Monitoring the Quality of Medical News Stories

In recent years, sites that monitor the completeness and accuracy of medical news reporting have been established in Australia (http://www.mediadoctor.org.au), Canada (http://www.mediadoctor.ca), Hong Kong (http://www.mediadoctor.hk), and the US (http://www.healthnewsreview.org). To address the question posed in this Policy Forum, we accessed and analysed data from the Media Doctor Australia site. This site posts reviews of health news stories published in the Australian commercial and publicly funded media, including newspapers, online news, television, and radio broadcast transcripts [5],[6],[12]. The focus is on stories that make therapeutic claims about new treatments and procedures, including diagnostic tests. News stories are not limited to local content, and include “wire” stories from major news organizations and stories from overseas media outlets that are carried by Australian media. The stories are identified from regular searches of a wide range of online news Web sites, along with media releases, journal articles, and other material relevant to the stories. Two raters independently score each news story according to ten criteria (See Box 1 for a description of rating criteria and Media Doctor Australia methods).

**Box 1. Media Doctor Rating Criteria**

Criteria reflect the extent to which the story:

1. Reported on the novelty of the intervention
2. Reported on the availability of the intervention
3. Described any treatment or diagnostic options available
4. Avoided elements of disease mongering
5. Reported on evidence supporting the intervention
6. Quantified the benefits of intervention
7. Described the harms of intervention
8. Reported on the costs of intervention
9. Consulted with independent expert sources
10. Did not rely heavily on a media release

* Each story was sent to two of 15 reviewers, comprising clinicians, public health specialists, medical writers, journalists, and clinical and population health researchers, who conducted the evaluations in a voluntary capacity. Reviewers rated stories about medical interventions and diagnostic tests independently, using validated rating instruments and rating guides [12]. The instruments contain 10 items (see above) that are identical to those used by the sister sites “Media Doctor Canada” and “Health News Review” in the United States [4],[7]. Scores are assigned by each reviewer based on a scoring guide. Reviewers put their draft reviews in a password-protected area of the Web site and any discrepancies are resolved by consensus. If necessary, a third reviewer is used to settle disagreements. To ensure objectivity and reliability, all reviews are screened by a researcher, who checks the scores and edits comments. Scores are “satisfactory,” “not satisfactory,” or “not applicable.” Both reviewers contribute to a comments section, which is used to highlight the strengths of the story or aspects that could have been improved and areas that are not covered in the rating instrument, such as the use sensationalist language or inappropriate headlines.

## Categorising the Authorship of Health News Reports

There has been little empirical research on the relationship between the authorship of articles and the content and quality of the stories. Anecdotally, specialist health journalists can provide lucid and succinct summaries of complex research, which can inform both the public and the researcher community. In operating the media monitoring sites, we have avoided naming specific journalists, preferring to concentrate on reporting the performance of the media outlets. We examined the provenance of 1,337 medical news stories published by the Australian mainstream media between 2004 and 2009, and subsequently rated by Media Doctor Australia. Although journalists are not named on the Web site, author information is recorded in a password-protected area of the Media Doctor database. Based on the “bylines” (who wrote the story) we placed the authors into six categories (Box 2).

**Box 2. Categorisation of Journalists Used in This Report**

1. *No byline*: All articles that did not identify authors
2. *General journalist*: A Google search on the author's name revealed no reporting specialty
3. *Overseas media*: Story imported from an overseas media outlet (e.g., New York Times)
4. *News organizations*: Story bought from a news syndicate, such as Associated Press or Reuters
5. *Health journalist*: A Google search identified the author as being a “health,” “medical,” or “science” reporter
6. *Specialist health journalist*: A Health Journalist subcategory in which the journalist had 10 or more stories posted on the Media Doctor web site during the period of the study

## Relationship between Categories of Journalists, Media Outlets, and the Quality of the Stories

The key issue was whether the more experienced specialist health journalists wrote stories of higher quality than journalists in the other categories. In making this judgement we were aware that the media outlet where the journalist worked was a potential confounder; even the best health journalist can have a story ruined by inappropriate editing or production. The 1,337 stories were published by 12 Australian media outlets between February 2004 and March 2009 (Table 1). Three hundred twenty stories had no byline. Of the remainder, 193 were written by 143 nonspecialist journalists; 415 came from four news organizations (Australian Associated Press [AAP], Associated Press [AP], Agence France Presse [AFP], and Reuters) and 39 came from 12 foreign media outlets (including ABC, BBC, Boston Globe, Guardian, The New York Times, The Telegraph UK, The Times, Los Angeles Times, Washington Post); 142 stories were written by 65 health/science journalists, and 228 stories were written by eight specialist health journalists, all based in Australia.

**Table 1:** Average unadjusted scores for medical news stories written by different categories of journalists and sourced from different news organisations.

Source	*n*	Average Score*	95% CI
***Category of Journalist***			
**No byline**	320	44.1	41.8, 46.4
**General journalist**	193	44.8	41.7, 47.9
**Overseas media**	39	50.6	43.9, 57.3
**News organizations**	415	54.9	53.0, 56.7
**Health journalists**	142	56.2	52.8, 59.7
**Specialist health journalists**	228	59.6	56.7, 62.6
***Australian Media Outlet***			
**Broadsheet newspapers**			
**Sydney Morning Herald (Sydney)**	252	58.8	56.3, 61.3
**The Australian (National)**	256	57.9	55.4, 60.5
**The Age (Melbourne)**	96	55.0	51.0, 59.0
**The West Australian (Perth)**	11	50.7	31.3, 50.7
**Tabloid newspapers**			
**The Sun Herald (Sydney)**	14	53.7	43.6, 63.8
**The Daily/Sunday Telegraph (Sydney)**	68	52.0	46.6, 57.4
**The Herald Sun (Melbourne)**	58	43.7	37.7, 49.6
**The Courier Mail (Brisbane)**	15	41.5	31.3, 51.6
**Internet news sites**			
**NineMSN (National: private)**	247	51.4	48.9, 53.9
**Australian Broadcasting Corporation (National: public)**	242	45.2	42.5, 48.0
**TV current affairs programs**			
**A Current Affair (Channel 9; national)**	30	34.7	27.3, 42.0
**Today Tonight (Channel 7; national)**	48	32.7	26.9, 38.6

a We assessed the quality of each news story using the Media Doctor criteria (Box 1), where total scores (expressed as proportion of all items that were rated “satisfactory”) were derived for stories that had seven or more items rated either satisfactory or not satisfactory. Stories with fewer than seven completed items were excluded. The data presented in this table are unweighted mean scores with their 95% confidence intervals.

The media outlets that published these stories are summarised in Table 1, which also presents the mean scores by media outlet and journalist category. The mean scores were highest for the broadsheet newspapers and lowest for the human interest current affairs programs. The difference between the average scores of the highest and lowest performing media outlets was 26.1% (95% CI 19.9%, 32.2%). The variation in unadjusted scores between the highest and lowest performing categories of journalists was less—a range of 15.5% (95% CI 11.2%, 19.8%) (Table 2). To deal with potential confounding by media outlet, we adjusted the analyses that compared average scores by categories of journalists, and these are given in Table 2. Using stories published without a byline as the reference category, those with significantly higher average scores were from news organizations, science/health journalists and specialist health journalists; the latter scored highest.

**Table 2:** Comparisons of Media Doctor scores for stories written by different categories of journalists.

Comparison	Mean Difference	95% CI^a^	*p*-Value*
**No byline (** ***n*** ** = 320)**	Reference	Reference	Reference
**General journalist (** ***n*** ** = 193)**	0.1	−3.9, 4.1	0.954
**Overseas media (** ***n*** ** = 39)**	1.2	−5.7, 8.1	0.730
**News organization (** ***n*** ** = 415)**	7.5	4.3, 10.7	<0.001
**Health journalist (** ***n*** ** = 142)**	8.1	3.8, 12.4	<0.001
**Specialist health journalist (** ***n*** ** = 228)**	12.5	8.9, 16.2	<0.001

See Box 2 for definitions.

a Our main hypothesis was that stories written by specialist health journalists would have higher scores than those from other sources. In the analysis the association between author category and score was examined, using stories without by-lines as the reference group. Comparisons were made between author types using a generalised linear mixed model. The outcome variable in the model was the article score, the predictor variable of interest was author category, which was included as a factor, and media outlet was used as the random effect.

Of the large news organizations examined, the company with the highest average scores was AP (Table 3). Differences between AP and other news organizations ranged from 7% to 15%, but after adjustment for multiple comparisons the only statistically significant difference was that between AP and AFP, 15.3% (95% CI 2.9% to 27.7%).

**Table 3:** Comparisons of Media Doctor scores for stories sourced from different news organisations.

Comparison	Mean Difference	95% CI^a^	*p*-Value*
**AFP versus AP**	−15.3	−27.7, −2.9	0.008
**AFP versus Reuters**	−8.2	−17.1, 0.7	0.086
**AAP versus AP**	−9.2	−19.3, 0.9	0.088
**AAP versus AFP**	6.1	−2.5, 14.8	0.263
**AP versus Reuters**	7.2	−3.1, 17.5	0.278
**AAP versus Reuters**	−2.0	−7.3, 3.2	0.749

a Adjusted for multiple comparisons using the Tukey-Kramer procedure.

## How Should We Interpret These Results?

It does matter who writes news stories that cover the benefits and harms of health care interventions. Stories written by specialist health journalists were superior to those written by other groups. These data illustrate what can be achieved in terms of high-quality health news reporting, but this ideal is seldom reached. The analyses also underscore the importance of which outlets journalists work for. Traditional broadsheet newspapers scored highest and commercial human interest programs consistently returned the poorest scores. We presume that these differences reflect not only the professional skills of journalists, but also editorial policies, which dictate the target audience, the writing style (favouring human interest over evidence), the length of the article, and the extent to which it serves particular sectoral interests (e.g., a patient support group or identifiable victims of a disease). These findings are not surprising, but some of the differences were large and likely to translate into flawed information for consumers, with an adverse effect on health literacy.

These findings are also significant because financial pressures in the industry threaten the jobs of experienced health journalists and the future of broadsheet newspapers. The internet has become a formidable rival to the more traditional forms of news [13]. Newspaper circulation has fallen and some experts envisage their end within a decade [14]. While this is speculation, there is no doubt that the traditional media are in a state of flux and there is pressure to economise. One outcome has been the downsizing of newsrooms across the world. An easy option for a pressured editor is to purchase health stories from foreign media outlets or news organisations. But the data presented here suggest that s/he should choose carefully. AP achieved fairly high and consistent ratings, whereas AFP had significantly lower average scores.

## What Are the Policy Implications of These Results?

Obvious suggestions to improve health reporting include better training of all journalists about evidence-based medicine during their undergraduate education. Major outlets could invest in more specialist health journalists and rely on fewer imported health stories. However, each of these suggestions comes at a cost, which may be substantial and unsustainable for the foreseeable future.

Another solution is to demand more responsibility from researchers and their institutions when interacting with the media. Research funding bodies usually require grant applicants to describe how they will disseminate their findings. They should ensure that information given to the media through press releases and comments is accurate and balanced. This role properly lies with the principal investigators, but funding bodies, research institutions, universities, those responsible for media promotion, and journals publishing the work share the responsibility to make more balanced claims about the findings, their importance and implications. The intention has to be promotion of the findings of good science, not self-promotion by researchers, sponsors, institutions, or journals, which all stand to benefit from media coverage. The public deserves to be well-informed about the research it funds. While we may not be able to directly influence which journalist writes health stories, researchers can make it easier for less-experienced journalists to do a good job. Better collaboration of researchers and health professionals with journalists and news outlets is an important step towards more objective communication.

## Abbreviations

AAP: Australian Associated Press
AFP: Agence France Presse
AP: Associated Press

## References

[1] Tian Y, Robinson JD (2008). Incidental health information use and media complementarity: a comparison of senior and non-senior cancer patients.. Patient Educ Couns.

[2] Viswanath K, Breen N, Meissner H, Moser RP, Hesse B (2006). Cancer knowledge and disparities in the information age.. J Health Commun.

[3] Kovic I, Lulic I, Brumini G (2008). Examining the medical blogosphere: an online survey of medical bloggers.. J Med Internet Res.

[4] Cassels A, Lexchin J (2008). How well do Canadian media outlets convey medical treatment information? Initial findings from a year and a half of media monitoring by Media Doctor Canada.. Open Medicine.

[5] Wilson A, Bonevski B, Jones A, Henry D (2009). Media reporting of health interventions: signs of improvement, but major problems persist.. PLoS ONE.

[6] Bonevski B, Wilson A, Henry DA (2008). An analysis of news media coverage of complementary and alternative medicine.. PLoS ONE.

[7] Schwitzer G (2008). How do US journalists cover treatments, tests, products, and procedures? An evaluation of 500 stories.. PLoS Med.

[8] Chapman S, Nguyen TN, White C (2007). Press-released papers are more downloaded and cited.. Tob Control.

[9] Ransohoff DF, Ransohoff RM (2001). Sensationalism in the media: when scientists and journalists may be complicit collaborators.. Eff Clin Pract.

[10] Hodgetts D, Chamberlain K, Scammell M, Karapu R, Waimarie Nikora L (2008). Constructing health news: possibilities for a civic-oriented journalism.. Health (London).

[11] Larsson A, Oxman AD, Carling C, Herrin J (2003). Medical messages in the media—barriers and solutions to improving medical journalism.. Health Expect.

[12] Smith DE, Wilson AJ, Henry DA (2005). Monitoring the quality of medical news reporting: early experience with media doctor.. Med J Aust.

[13] Fitzgerald T (2009). Newspaper circulation drops 11 percent.. Media Life..

[14] Simmons M (2009 16 September). Web expert tells Fairfax: newspapers have 10 years. Tops. Crikey.. http://www.crikey.com.au/2009/09/16/web-expert-tells-fairfax-newspapers-have-10-years-tops/.

